# Impact of early death recording on international comparison of acute myocardial infarction mortality – administrative hospital data study using the example of Germany and the United States

**DOI:** 10.1186/s12913-024-11044-6

**Published:** 2024-05-07

**Authors:** Ulrike Nimptsch, Thomas Mansky, Reinhard Busse

**Affiliations:** 1 Department of Health Care Management, Technische Universität Berlin, Straße des 17. Juni 135, 10623 Berlin, Germany; 2 Dovestr. 11, 10587 Berlin, Germany

**Keywords:** Acute myocardial infarction, Hospital, Quality indicator, Mortality, Administrative data

## Abstract

**Background:**

In-hospital mortality from acute myocardial infarction (AMI) is widely used in international comparisons as an indicator of health system performance. Because of the high risk of early death after AMI, international comparisons may be biased by differences in the recording of early death cases in hospital inpatient data. This study examined whether differences in the recording of early deaths affect international comparisons of AMI in-hospital mortality by using the example of Germany and the United States, and explored approaches to address this issue.

**Methods:**

The German Diagnosis-Related Groups Statistics (DRG Statistics), the U.S. National Inpatient Sample (NIS) and the U.S. Nationwide Emergency Department Sample (NEDS) were analysed from 2014 to 2019. Cases with treatment for AMI were identified in German and U.S. inpatient data. AMI deaths occurring in the emergency department (ED) without inpatient admission were extracted from NEDS data. 30-day in-hospital mortality figures were calculated according to the OECD indicator definition (unlinked data) and modified by including ED deaths, or excluding all same-day cases.

**Results:**

German age-and-sex standardized 30-day in-hospital mortality was substantially higher compared to the U.S. (in 2019, 7.3% vs. 4.6%). The ratio of German vs. U.S. mortality was 1.6. After inclusion of ED deaths in U.S. data this ratio declined to 1.4. Exclusion of same-day cases in German and U.S. data led to a similar ratio.

**Conclusions:**

While short-duration treatments due to early death are generally recorded in German inpatient data, in U.S. inpatient data those cases are partially missing. Excluding cases with short-duration treatment from the calculation of mortality indicators could be a feasible approach to account for differences in the recording of early deaths, that might be existent in other countries as well.

**Supplementary Information:**

The online version contains supplementary material available at 10.1186/s12913-024-11044-6.

## Background

Acute myocardial infarction (AMI) is a common emergency condition in western industrialized countries. Patient survival depends on timely treatment, in the case of ST-elevation myocardial infarction (STEMI) preferably by means of percutaneous coronary intervention (PCI) [1]. This is why in-hospital mortality for AMI is widely used in international comparisons as an indicator of health system performance that allows conclusions to be drawn about the effectiveness of care processes [2]. 

Measurement of AMI in-hospital mortality often relies on administrative hospital data, which are generated through the processes of hospital care. For instance, the AMI mortality indicators of the OECD Healthcare Quality and Outcomes programme, previously known as Healthcare Quality Indicators (HCQI) Project, are based on administrative hospital data from the respective member states [3]. 

The HCQI comparison of admission-based age-and-sex standardized in-hospital mortality for AMI shows considerable variation across countries: While in the year 2019 the United States of America (U.S.) rate of 4.9% was below the OECD average of 6.6%, in Germany 8.3% of AMI patients died after admission [4]. These figures might reflect subpar AMI care in Germany and there might be potential for improvement [5]. However, compared to other countries, the German health system is characterized by high hospital capacity and easy access to immediate hospital services [4, 6]. In 2019 more than 80% of AMI patients in Germany received coronary angiography or PCI in the first treating hospital [7]. 

As mentioned by the OECD, differences in administrative data-based AMI mortality figures between countries might not solely reflect differences in health system performance, but as well differences in length of stay, transfers to other hospitals, or disease severity [4]. Yet, there might be another source of bias which has not been comprehensively explored, namely, whether short treatments due to early death after arrival to the hospital are completely captured in inpatient databases. This may vary in different countries, depending on billing practices within the respective health system. Regarding AMI mortality this issue is of relevance, since fatal events may occur in the emergency department (ED) before the patient is admitted to a specialized hospital ward. A German clinical registry reported that almost one third of deaths in hospitalized patients diagnosed with AMI occurred during the first 24 h after onset of symptoms [8]. A more recent study from the U.S. based on administrative data found that about 44% of patients with AMI and cardiogenic shock died within two days of admission [9]. 

In Germany, treatments of patients who died shortly after arrival to the hospital are normally billed as inpatient cases via the all-payer German Diagnosis Related Groups System. Although this practice has been subject to legal disputes between hospitals and statutory health insurance funds in the past, it has been confirmed by German case-law several times [10, 11]. This implies that short-duration treatments due to early death are virtually completely recorded in German inpatient data. A recent study of combined ED, inpatient, and outpatient data of 16 large German hospitals found that the percentage of early fatalities with outpatient billing was less than 1% [12].

In the U.S., treatments of patients who died in the ED might be billed as outpatient if they were not admitted to a hospital ward before death. Regarding Medicare beneficiaries, the Centers for Medicare & Medicaid Services (CMS) specified in 2013 that cases should be billed as inpatient if the patient is expected to require a hospital stay that crossed two midnights. This also includes stays in which this expectation is supported, but the length of the actual stay was less than two midnights due to death [13]. However, this rule seems to be subject to debate, in particular regarding the case of AMI [14]. Moreover, a majority of the U.S. population receives their coverage from private health insurance [15], and billing practices regarding inpatient or outpatient payment may vary by provider [16]. Therefore, in contrast to Germany, treatments for AMI followed by early death might be less completely recorded in U.S. inpatient data.

The aim of this study was to investigate whether early death recording affects the comparison of AMI in-hospital mortality by using the example of Germany and the U.S., and to explore approaches to account for this issue by modifying mortality indicator definitions. Beyond that, the analysis provides a cross-country comparison of AMI population characteristics and patterns of care.

## Methods

### Aim, design, and setting of the study

The aim of this study was to investigate the impact of early death recording on international comparison of acute myocardial infarction mortality based on hospital administrative data. Within an observational study design national in-hospital mortality for AMI was compared between Germany and the U.S. Different modifications to the composition of mortality figures were applied to administrative data from acute inpatient and emergency department care of both countries.

### Data

For Germany, the Diagnosis-Related Group Statistics (DRG Statistics) from 2014 to 2019 were analysed. The DRG Statistics are a national complete all-payer database comprising data records of all inpatient stays in all German acute care hospitals, except for psychiatric and psychosomatic treatment. The data contain principal and secondary diagnoses that are coded according to the German modification of the International Classification of Diseases (ICD-10-GM). Procedures are coded according to the German procedure coding system (Operationen- und Prozedurenschlüssel, OPS). Information on sex, age, source of admission, discharge disposition, and length of stay are also included. The data were provided by the Research Data Centre of the German Federal Statistical Office after the application for data use was approved, and were accessed via remote execution [17].

For the U.S., the National Inpatient Sample (NIS) and the Nationwide Emergency Department Sample (NEDS) of the Health-Care Cost and Utilization Project (HCUP) from 2014 to 2019 were analysed. HCUP is sponsored by the Agency for Healthcare Research and Quality (AHRQ). NIS is an all-payer sample of inpatient stays from all hospitals participating in HCUP, covering about 7 million inpatient stays per year [18]. NEDS is an all-payer ED database, covering about 30 million ED visits with or without inpatient admission per year [19]. Diagnosis coding in both datasets changed from ICD-9 Clinical Modification (ICD-9-CM) to ICD-10-CM in the year 2015. The first listed diagnosis in an inpatient record or an ED record represents the principal diagnosis. Procedures are coded according to the ICD-9 procedure coding system (PCS), or ICD-10-PCS, respectively. In NEDS data, procedures undertaken during ED visits without inpatient admission are coded according to the Clinical Classifications Software (CCS) services and procedures classification. Both datasets contain information on sex, age, and discharge disposition. Information on length of stay and source of admission are only available for inpatient cases. Both, NIS, and NEDS represent approximately 20% of inpatient stays or, respectively, ED visits in U.S. hospitals. Weights are available to calculate estimates for the entire U.S. population. NIS and NEDS data were provided by the HCUP Central Distributor after the application for data use was approved.

### Participants

In the German DRG Statistics, as well as in the U.S. NIS, inpatient cases with treatment for AMI were identified by applying the inclusion criteria of the OECD indicator AC2 “AMI 30 day in-hospital (same hospital) mortality using unlinked data (admission based)”. The indicator covers hospital admissions for acute care with a principal diagnosis of AMI of patients aged 15 years and older. All admissions (including day cases) are to be counted in the denominator including cases transferred-in from another hospital, as well as cases transferred-out to another hospital. The numerator of this indicator comprises deaths in the same hospital that occurred within 30 days of the admission date [20]. Additionally, the OECD indicator definition requires a restriction to cases with emergency treatment. However, this requirement could not be met, as German data allows no valid separation of emergency from elective, or non-urgent status. Although German data contain a flag for ‘emergency’ admission, this flag actually identifies cases without referral by a resident physician. So, this flag rather depicts the administrative mode of access than clinical emergency. On the other hand, U.S. NIS data contain a flag for elective admission. Initial analysis of NIS data revealed that the proportion of AMI cases coded as elective was less than 5% and in-hospital mortality of those cases (4.3%) was only slightly deviant from the mortality of the whole AMI inpatient population (4.6%). Therefore, it was decided to keep all cases in the analysis of German and U.S. data, regardless of emergency or elective status.

Aiming to identify AMI cases that are not recorded in U.S. inpatient data, ED deaths without inpatient admission of patients with a first listed diagnosis of AMI aged 15 years and older were extracted from NEDS data. AMI cases transferred-out from the ED to another hospital were not considered, as those cases will likely appear in inpatient data after admission to the designated hospital. Cases that were released alive from the ED without inpatient admission were not considered, because in those cases the diagnosis of AMI might rather represent a ruled-out diagnosis [21]. Details of inclusion and exclusion criteria are given in supplementary material 1. Case selection flow is displayed in supplementary material 2.

### Analysis

While German data represent a full sample of all inpatient cases in German hospitals, national numbers of U.S. inpatient cases and ED visits were estimated by using the respective weights provided by HCUP.

Characteristics of cases were analysed descriptively. Definitions of presented variables are displayed in supplementary material 1. Aiming to explore possible differences in rates of hospitalizations for AMI that are not related to demographic differences, German and U.S. population-based AMI case rates were standardized by 5-year age groups and sex according to the OECD 2010 standard population [22].

To assess differences in patterns of care and mortality, relative distributions of cases and deaths were stratified by length of stay. As no time stamp is available in U.S. data and the validity of time stamps in German data is uncertain [23], length of stay was determined by admission date. Cases with a length of stay of 0 days (i.e., discharge date - admission date = 0) were determined as same-day stays. As in U.S. data no information on length of stay is available for ED visits without inpatient admission, ED death cases were assigned to a length of stay less than one day and thus determined as same-day stays.

Age-and-sex specific 30-day in-hospital mortality rates were displayed for all AMI cases, as well as for the subgroup of cases with transmural/ST-elevation myocardial infarction (STEMI).

AMI 30-day in-hospital mortality was calculated for each year of observation. German mortality figures were standardized by 5-year age groups and sex, according to the U.S. AMI disease population of the respective calendar year. According to the definition of the OECD indicator, mortality figures refer to cases aged 45 years and above.

To assess the possible bias resulting from differences in the recording of early deaths, deliberate modifications were applied to the composition of mortality figures. In a first step, U.S. mortality figures were recalculated after adding ED deaths without admission to the denominator and the nominator. In a second step, German and U.S. mortality figures were recalculated after excluding all same-day stay cases from the denominator and the nominator. The analyses were repeated for the subgroup of STEMI cases.

Differences between German and U.S. 30-day in-hospital mortality figures were expressed as ratios with 95% confidence intervals. Those were calculated based on confidence intervals for German age-sex standardized rates, and confidence intervals for U.S. rates under consideration of the sampling design, respectively [24]. 

The analyses were conducted using SAS Version 9.4 (SAS Institute Inc., Cary, NC, U.S.A). Reporting adheres to the RECORD (Reporting of studies Conducted using Observational Routinely-collected health Data) Statement [25]. 

## Results

In Germany, 1.30 million hospitalizations for acute myocardial infarction were observed from 2014 to 2019. In the U.S., 3.88 million AMI hospitalizations were estimated during this time span. After consideration of about 25,600 cases with a first listed diagnosis of AMI who died in the ED without inpatient admission, the estimated number of AMI cases in the U.S. was 3.90 million. After age-sex standardization to the 2010 OECD standard population average annual AMI hospitalization rates per 100,000 population in Germany (235) and the U.S. (236) were quite similar (Table 1).

**Table 1:** Characteristics of AMI cases treated for acute myocardial infarction, 2014 to 2019

	Germany (full sample)	United States (estimates weighted to national average)		
	DRG statistics	National Inpatient Sample (NIS)	Nationwide Emergency Department Sample (NEDS)	NIS and NEDS combined
	*Inpatient treatment * *(including early deaths)*	*Inpatient treatment*	*ED death without admission*	*Inpatient treatment or ED death without admission*
Cases with a principal or first listed diagnosis of AMI N (%)	1,300,718 (100.0)	3,875,610 (100.0)	25,598 (100.0)	3,901,208 (100.0)
Per 100,000 population per year	262.8	199.3	1.3	200.6
Per 100,000 population per year, age-and-sex standardized ^a^	235.1	234.5	1.6	236.1
Female N (%)	433,530 (33.3)	1,467,585 (37.9)	10,553 (41.2)	1,478,138 (37.9)
Age > = 65 years N (%)	847,114 (65.1)	2,209,870 (57.0)	17,860 (69.8)	2,227,729 (57.1)
Age Median (P25 – P75)	72 (60–80)	67 (57–77)	72 (62–83)	67 (57–77)
Transmural/ST-elevation AMI N (%)	422,633 (32.5)	1,001,485 (25.8)	14,100 (55.1)	1,015,585 (26.0)
Cardiogenic shock N (%)	85,258 (6.6)	240,130 (6.2)	2,427 (9.5)	242,557 (6.2)
Resuscitation (%)	71,798 (5.5)	71,640 (1.8)	13,313 (52.0)	84,953 (2.2)
Percutaneous coronary intervention N (%)	766,497 (58.9)	1,835,440 (47.4)	1,714 (6.7)	1,837,154 (47.1)
Coronary artery bypass graft N (%)	71,597 (5.5)	338,870 (8.7)	<=10 (0.0)	<=338,880 ( < = 8.7)
Transferred-in from other acute care hospital N (%)	175,698 (13.5)	695,220 (17.9)	n/a	695,220 (17.8)
Treated in emergency department N (%)	n/a	2,811,935 (72.6)	25,598 (100.0)	2,837,533 (72.7)
Same-day stay N (%) ^b^	82,033 (6.3)	118,010 (3.0)	25,598 (100.0)	143,608 (3.7)
Length of stay Median (P25 – P75)	6 (3–9)	2 (1–5)	n/a	2 (1–5)
Transferred-out to other acute care hospital N (%)	215,728 (16.6)	298,680 (7.7)	n/a	298,680 (7.7)
Died in same hospital N (%)	107,741 (8.3)	182,340 (4.7)	25,598 (100.0)	207,938 (5.3)
Died in same hospital within 30 days N (%)	104,831 (8.1)	179,730 (4.6)	25,598 (100.0)	205,328 (5.3)

AMI: acute myocardial infarction; ED: emergency department; n/a: not available. ^a^ Directly standardized by sex and 5-year age-groups according to the 2010 OECD standard population, age > = 15. ^b^ As in U.S. NEDS data no information on length of stay is available for ED visits without inpatient admission, ED deaths without admission were assigned to a length of stay < 1 day (same-day stay)

Over time, age-and-sex standardized AMI hospitalization rates declined in Germany from 246 to 225, while the U.S. rate of 234 in 2014 was transiently elevated in 2016 and 2017, followed by a subsequent decline to 232 cases per 100,000 population in 2019 (supplementary material 3).

Compared to the U.S., the percentage of females among AMI cases was lower in Germany (33% vs. 38%) and median age was higher (72 vs. 67). In Germany, the percentage of STEMI was higher (33% vs. 26%), and a higher percentage of cases received PCI (59% vs. 47%) while coronary artery bypass surgery was less frequently performed (6% vs. 9%). Cardiopulmonary resuscitation was more often coded in Germany (5.5%) than in the U.S. (2.2% after consideration of ED deaths, Table 1).

**Fig. 1:**
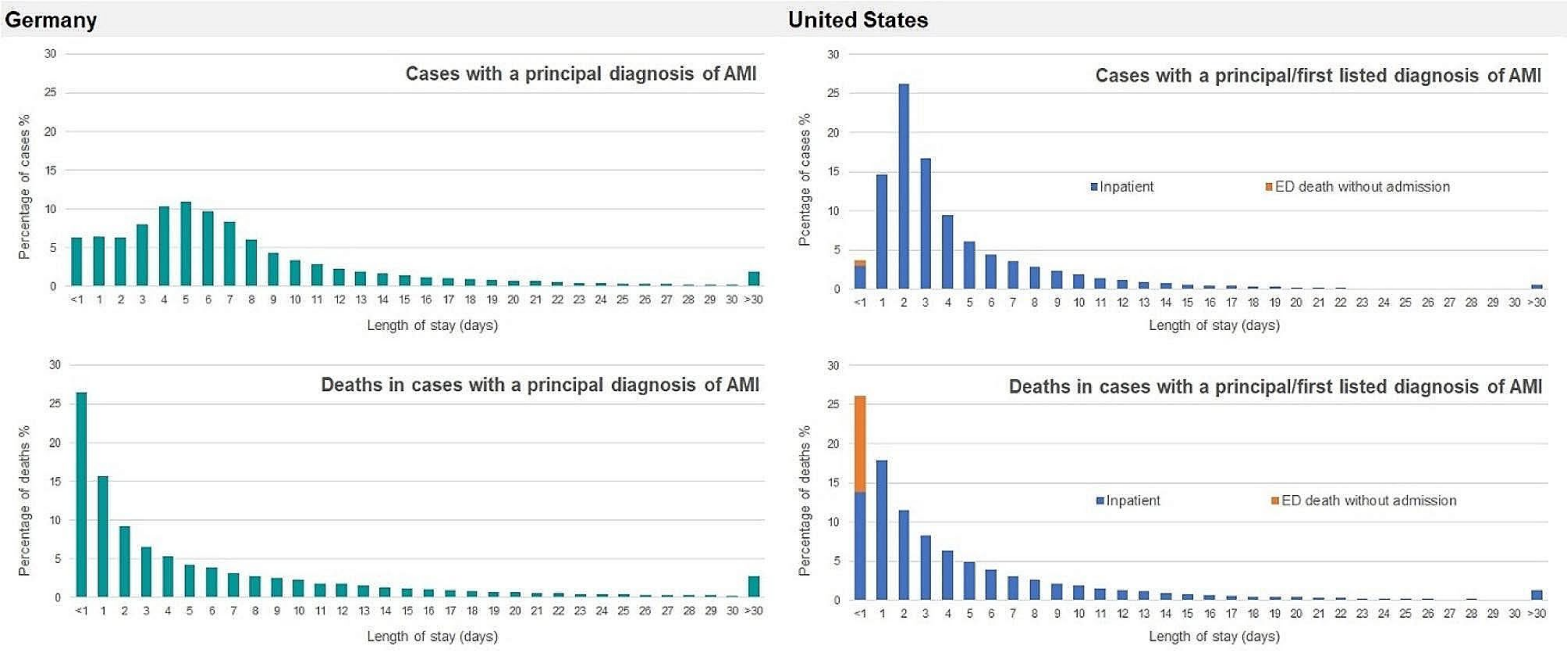
Relative distribution of AMI cases and AMI in-hospital deaths by length of stay, 2014 to 2019 AMI: acute myocardial infarction; ED: emergency department. Note: As in U.S. NEDS data no information on length of stay is available for ED visits without inpatient admission, ED deaths without admission were assigned to a length of stay < 1 day (same-day stay)

The relative distribution of cases by length of stay (LOS) revealed different patterns. While in the U.S. there was a marked peak at a LOS of two days, in Germany LOS was more broadly distributed around five days (Fig. 1). The relative distribution of deaths showed that in Germany more than one quarter of deaths of patients diagnosed with AMI occurred at the admission date. In U.S. data this proportion was quite similar after considering ED deaths without admission as same-day stays (Fig. 1).

**Fig. 2:**
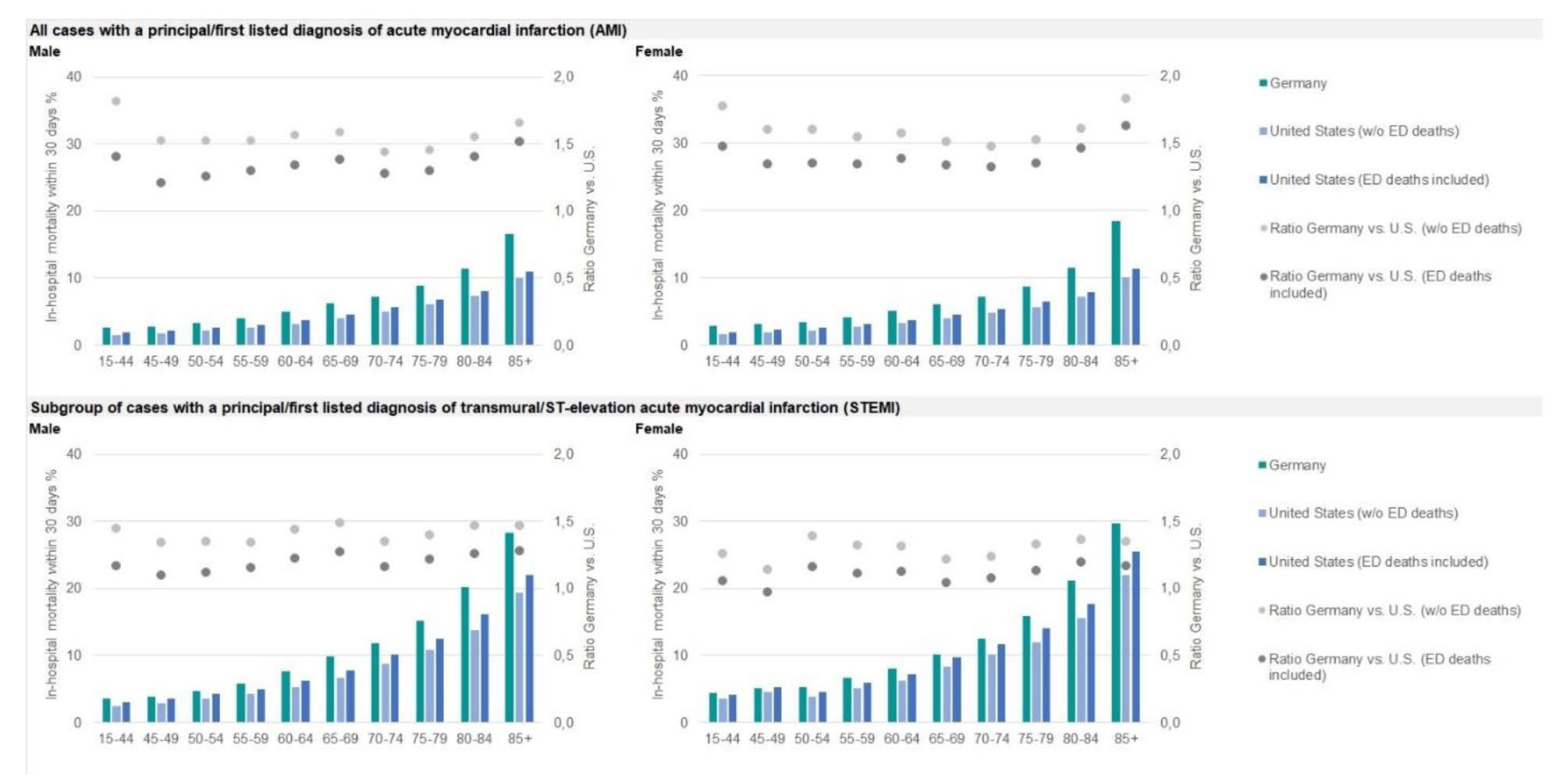
AMI 30-day in-hospital mortality stratified by sex and age groups, 2014 to 2019 AMI: acute myocardial infarction; ED: emergency department

Age-and-sex specific 30-day in-hospital mortality rates showed that mortality in Germany was substantially higher throughout all age groups. Regarding the whole AMI population those differences were most pronounced in women aged 85 years and above. Considering ED deaths in U.S. data reduced, but not diminished age-and-sex specific mortality differences. After restriction to the subgroup of cases with STEMI the mortality differences between Germany and the U.S. were smaller, but still existent in most age-sex groups (Fig. 2).

Stratification by characteristics and treatment variables revealed higher German 30-day in-hospital mortality in all strata. The mortality difference was most pronounced in cases with cardiogenic shock (Germany 53% vs. U.S. 33% after consideration of ED deaths). Considering ED deaths in U.S. data reduced the mortality difference in strata of age, sex, STEMI, and cardiogenic shock, but not in strata of PCI and CABG. Considering ED deaths in U.S. data reversed the mortality difference in same-day cases, and cases with resuscitation (see supplementary material 4).

**Table 2:** 30-day in-hospital mortality of AMI cases, 2014 to 2019

	2014	2015	2016	2017	2018	2019
**All cases with acute myocardial infarction (AMI)**						
**AMI 30-day mortality in same hospital**						
Germany (standardized to U.S. disease population) %	8.0	7.8	7.6	7.5	7.6	7.3
United States (inpatient data only) %	5.1	4.9	4.8	4.8	4.7	4.6
*Ratio Germany vs. United States*	*1.6*	*1.6*	*1.6*	*1.6*	*1.6*	*1.6*
*[95% confidence interval]*	*[1.5–1.6]*	*[1.5–1.7]*	*[1.5–1.6]*	*[1.5–1.6]*	*[1.6–1.7]*	*[1.5–1.7]*
**AMI 30-day mortality in same hospital. ED deaths considered in US data**						
Germany (standardized to U.S. disease population) %	8.0	7.8	7.6	7.5	7.6	7.3
United States (including ED deaths without admission) %	5.9	5.7	5.4	5.4	5.2	5.2
*Ratio Germany vs. United States*	*1.4*	*1.4*	*1.4*	*1.4*	*1.5*	*1.4*
*[95% confidence interval]*	*[1.3–1.4]*	*[1.3–1.4]*	*[1.3–1.4]*	*[1.3–1.5]*	*[1.4–1.5]*	*[1.4–1.5]*
**AMI 30-day mortality in same hospital. Same-day stays (LOS < 1) excluded**						
Germany (standardized to U.S. disease population) %	6.2	6.1	5.8	5.8	5.8	5.5
United States (inpatient data only) %	4.4	4.3	4.2	4.2	4.0	3.9
*Ratio Germany vs. United States*	*1.4*	*1.4*	*1.4*	*1.4*	*1.4*	*1.4*
*[95% confidence interval]*	*[1.3–1.5]*	*[1.4–1.5]*	*[1.3–1.5]*	*[1.3–1.4]*	*[1.4–1.5]*	*[1.3–1.5]*
**Subgroup of cases with transmural/ST-elevation (STEMI) myocardial infarction**						
**Transmural/STEMI 30-day mortality in same hospital**						
Germany (standardized to U.S. disease population) %	10.3	10.5	10.7	10.8	11.1	10.9
United States (inpatient data only) %	7.1	7.3	8.3	8.2	8.0	8.0
*Ratio Germany vs. United States*	*1.5*	*1.4*	*1.3*	*1.3*	*1.4*	*1.4*
*[95% confidence interval]*	*[1.4–1.6]*	*[1.3–1.5]*	*[1.2–1.4]*	*[1.2–1.4]*	*[1.3–1.5]*	*[1.3–1.5]*
**Transmural/STEMI 30-day mortality in same hospital. ED deaths considered in US data**						
Germany (standardized to U.S. disease population) %	10.4	10.6	10.8	10.9	11.1	11.0
United States (including ED deaths without admission) %	7.8	8.6	10.2	9.8	9.1	9.2
*Ratio Germany vs. United States*	*1.3*	*1.2*	*1.1*	*1.1*	*1.2*	*1.2*
*[95% confidence interval]*	*[1.2–1.4]*	*[1.2–1.3]*	*[1.0–1.1]*	*[1.1–1.2]*	*[1.2–1.3]*	*[1.1–1.3]*
**Transmural/STEMI 30-day mortality in same hospital. Same-day stays (LOS < 1) excluded**						
Germany (standardized to U.S. disease population) %	7.4	7.6	7.5	7.6	7.8	7.6
United States (inpatient data only) %	5.6	5.9	6.6	6.5	6.3	6.2
*Ratio Germany vs. United States*	*1.3*	*1.3*	*1.1*	*1.2*	*1.2*	*1.2*
*[95% confidence interval]*	*[1.2–1.4]*	*[1.2–1.4]*	*[1.1–1.2]*	*[1.1–1.3]*	*[1.1–1.3]*	*[1.1–1.3]*

ED: emergency department; LOS: length of stay. Note: According to the definition of the OECD indicator AC2 “AMI 30 day in-hospital (same hospital) mortality using unlinked data (admission based)” all figures refer to cases aged 45 years and above. German figures were standardized to the U.S. disease population of the respective calendar year

**Fig. 3:**
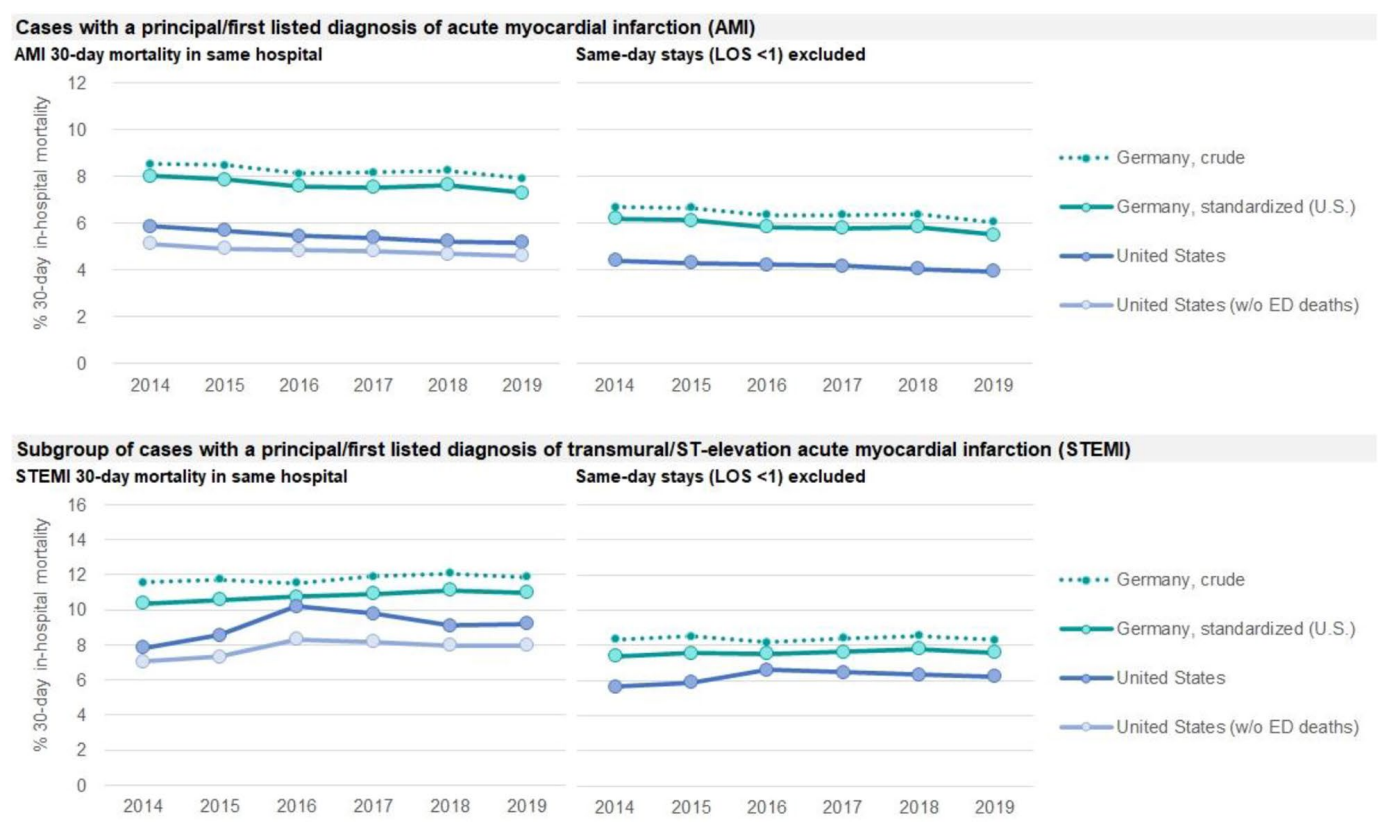
AMI 30-day in-hospital mortality by year, 2014 to 2019 AMI: acute myocardial infarction. LOS: Length of stay. Note: According to the definition of the OECD indicator AC2 “AMI 30 day in-hospital (same hospital) mortality using unlinked data (admission based)” all figures refer to cases aged 45 years and above. German figures were standardized to the US disease population of the respective calendar year

The comparison of 30-day in-hospital mortality figures according to the OECD indicator definition showed higher German age-and-sex standardized mortality, compared to the U.S. (in 2019 7.3% vs. 4.6%, Fig. 3). In both countries a decline was visible from 2014 to 2019. During this time span the ratio of German vs. U.S. mortality was stable at 1.6. After consideration of ED deaths in U.S. data this ratio declined to 1.4. Exclusion of cases with same-day stay led to a similar ratio (Table 2; Fig. 3).

In the subgroup of cases with STEMI/transmural infarction German age-and-sex standardized in-hospital mortality was higher than in the U.S. (in 2019 10.9% vs. 8.0%, Fig. 3). A slight increase of in-hospital mortality was observed over time in both countries. In U.S. data a transient elevation of mortality was found in 2016, the year after transition from ICD-9-CM to ICD-10-CM (Fig. 3). In 2019 the ratio of German vs. U.S. mortality was 1.4. After consideration of ED deaths in U.S. data this ratio declined to 1.2. Exclusion of cases with same-day stay led to a similar ratio (Table 2; Fig. 3).

## Discussion

In Germany as well as in the U.S., more than one quarter of deaths of patients diagnosed with AMI occurred within 24 h after arrival to the hospital, or in the ED before inpatient admission. While short-duration treatments due to early death are generally recorded in German inpatient data, in U.S. inpatient data those cases are partially missing. Consequently, the consideration of ED deaths without inpatient admission in U.S. data resulted in higher mortality figures in contrast to using only inpatient data. However, although the difference between German and U.S. mortality declined after considering ED deaths in U.S. data, German AMI 30-day in-hospital mortality remained substantially higher.

The results of this study suggest that international comparisons of AMI mortality figures can be biased by international differences in the recording of early deaths, as different reimbursement systems may cause a different administrative handling of short-stay cases. Consequently, such cases may not completely appear in inpatient data. Therefore, the approach of excluding cases with a short duration of treatment from the calculation of mortality figures was explored. The rationale was, focusing only those cases that are most likely completely recorded in inpatient data of each country might allow more reliable comparisons. In fact, the ratio between German and U.S. AMI mortality was quite similar after excluding same-day cases from the data of both countries, compared to the ratio after including ED deaths in U.S. data.

This study investigated the case of Germany and the U.S. because sufficient, comparable, and easily accessible data are available from both countries. Yet, differences in the recording of early deaths might be existent in other countries as well, in one way or another [26]. The HCQI expert group stated that indicators for international comparison should be defined in a way that indicator results reflect issues in quality of care rather than differences in non-quality-of-care reasons, such as data collection methodologies. On the other hand, indicator definitions should also be internationally feasible, i.e., data should be derived without substantial additional resources [27]. The approach of excluding same-day cases from the calculation of mortality figures might be a feasible way to account for international differences in the recording of early deaths without additional use of ED data. This approach might also reduce possible bias resulting from differences in the frequency of early transfers to a specialized hospital, or differences in diagnostic accuracy which might be more existent in cases with a short duration of treatment. However, additional research on data from other countries is needed to confirm the appropriateness of this approach. It also must be mentioned that excluding same-day cases requires that same-day cases can be identified in inpatient data. However, this information should be available in inpatient databases of most countries, as date of admission and date of discharge are likely to be documented for administrative purposes.

In addition to international comparisons, it should also be mentioned that incomplete recording of early deaths in inpatient data may affect the national use of AMI mortality in inpatient quality indicator systems, if data collection practice varies between hospitals.

Another finding of the present study is that German AMI 30-day in-hospital mortality measured from administrative data was substantially higher compared to the U.S., even after accounting for early death recording. High AMI in-hospital mortality in Germany has also been reported by international studies in the past [28], as well as rather low AMI mortality in the U.S [29]. Besides early death recording, one possible explanation for the high in-hospital mortality for AMI in Germany relates to lower pre-hospital mortality, compared to other countries [28]. However, this presumption is difficult to verify, as studies on pre-hospital AMI mortality are scarce, and often small-scaled. A German study reported that in Berlin 66% of AMI deaths occurred outside the hospital [30], while an earlier study from the U.S. reported a share of 60% pre-hospital AMI deaths in Worcester, Massachusetts [31]. In rural areas, which are more prevalent in the U.S. than in Germany [32], the share of pre-hospital AMI deaths might be even higher. However, a recent German study reported that higher death rates for AMI in German rural regions are primarily the result of a higher AMI incidence compared to urban regions, while possibly delayed emergency care pathways seemed to be less influential [33]. 

Another recent study raised differences in the share of inter-hospital-transfers as possible reason for differences in AMI mortality between countries: frequent transfers inflate the denominator and thus, lead to a lower calculated in-hospital mortality [26]. However, the difference in the share of inter-hospital-transfers observed in the present study was not pronounced enough to explain the mortality difference.

The remaining difference between German and U.S. 30-day AMI in-hospital mortality, after accounting for early death recording, might also be related to differences in the length of stay in hospital [29]. Considering the markedly lower length of stay in U.S. hospitals, it seems likely that more deaths following AMI occur after hospital discharge in the U.S., compared to Germany. This assumption is supported by recent OECD publications, which also report U.S. 30-day AMI mortality based on linked data. This figure was at 9.3% in the year 2020, compared to 4.9% when based on unliked data. For Germany, national 30-day AMI mortality figures based on linked data are not available to date. In other industrialized countries reporting both figures, the difference between mortality based on unlinked data and mortality based on linked data was not as pronounced as in the U.S. (e.g., 5.6% vs. 7.2% in France, 6.5% vs. 7.1% in Spain) [4]. 

Moreover, the present study revealed that AMI cases in Germany were older, less often female, and were more often diagnosed with STEMI compared to U.S. AMI cases. Demographic differences in the disease populations were corrected by age-and-sex standardization. Possibly, the additional consideration of type of AMI in the calculation of mortality figures might further enhance comparability, since the mortality difference between Germany and the U.S. in the subgroup of STEMI cases was smaller than in the whole AMI population. In general, AMI severity might differ between both countries. In Germany, a twofold higher resuscitation rate was observed, that might – along with the higher percentage of STEMI cases – reflect a case higher case severity in the German AMI population.

The descriptive analysis also revealed differences in patterns of care, such as a higher percentage of treatment with PCI in Germany, compared to the U.S. This might indicate better care according to treatment guidelines but could also be related to the higher share of STEMI cases in the German AMI population. In contrast, the percentage of treatment with coronary artery bypass graft (CABG) was lower in Germany, compared to the U.S.

The strength of this study is the analysis of complete national administrative hospital data from Germany, and large representative national samples of administrative hospital and ED data from the U.S. However, several limitations should be considered. First, administrative hospital data is collected for billing purposes, and differences in coding, reimbursement rules, and data collection practice may impair international comparisons [34, 35]. One of those issues is the recording of early deaths which was subject of this study. Second, the deliberate assignment of ED deaths in U.S. data to a length of stay of less than one day was done for practical reasons. However, it is possible that this assumption might not be correct in all cases. In this context, it should also be considered that length of stay was determined based on the admission date instead of exact admission time. This might have caused an incomplete separation of cases with an overnight stay of stay less than 24 h. Third, possible differences in coding due to different modifications of the ICD-10 should be considered. While the German Modification bases its classification of AMI on anatomic criteria (transmural vs. non-transmural infarction), the Clinical Modification used in the U.S. allows the electrophysiological distinction between ST-elevation and non-ST-elevation infarctions. In general, incentives for AMI diagnosis coding might differ between Germany and the U.S. As well, the transition from ICD-9 to ICD-10 coding that took place in the U.S. in 2015 should be considered. Finally, the present study focused only admission-based AMI mortality, i.e., only deaths occurring in the same hospital were captured. Deaths occurring after hospital discharge remained unobserved, since a unique patient identifier to link the data across relevant datasets was not available in the studied data of both countries.

## Conclusion

Indicators of health care performance allow to identify potential for improvement in individual countries and can meaningfully support health policy decisions. Because of their availability and completeness, administrative hospital data might be the most suitable data source for international comparisons. However, defining indicators that reflect quality of care while being robust against non-quality-of-care related differences is challenging. Excluding cases with short-duration treatment from the calculation of AMI mortality figures might be a feasible approach to correct for differences in the recording of early deaths, that might be existent in other countries as well.

### Electronic supplementary material

Below is the link to the electronic supplementary material.

Supplementary Material 1

## Data Availability

German inpatient data (DRG statistics, 10.21242/23141.2014.00.00.1.1.0 to 10.21242/23141.2019.00.00.1.1.1) are available for research purposes from the Research Data Centre of the Federal Statistical Office (https://www.forschungsdatenzentrum.de/en) upon application. U.S. inpatient data (NIS, http://www.hcup-us.ahrq.gov/nisoverview.jsp) and U.S. emergency department data (NEDS, https://www.hcup-us.ahrq.gov/nedsoverview.jsp) are available from the HCUP Central Distributor upon application (https://cdors.ahrq.gov/). Aggregated data are available from the corresponding author upon request.

## Abbreviations

AMI: Acute myocardial infarction
CABG: Coronary artery bypass graft
CCS: Clinical Classifications Software
CMS: Centers for Medicare & Medicaid Services
DRG: Diagnosis-Related Groups
ED: Emergency department
HCQI: Healthcare Quality Indicators
ICD-10-CM: International Classification of Diseases, 10th Revision, Clinical Modification
ICD-10-GM: International Classification of Diseases, 10th Revision, German Modification
ICD-9-CM: International Classification of Diseases, 9th Revision, Clinical Modification
LOS: Length of stay
NEDS: Nationwide Emergency Department Sample
NIS: National Inpatient Sample
OECD: Organisation for Economic Co-operation and Development
OPS: Operationen- und Prozedurenschlüssel
PCI: Percutaneous coronary intervention
PCS: Procedure coding system
STEMI: ST-elevation myocardial infarction

## References

[1] Ibanez B, James S, Agewall S (2018). 2017 ESC guidelines for the management of acute myocardial infarction in patients presenting with ST-segment elevation: the Task Force for the management of acute myocardial infarction in patients presenting with ST-segment elevation of the European Society of Cardiology (ESC). Eur Heart J.

[2] Kelley E (2007). Health, spending and the effort to improve quality in OECD countries: a review of the data. J R Soc Promot Health.

[3] OECD. OECD Health Statistics. 2020. Definitions, sources and methods. Accessed 13 Oct 2023. https://www.oecd.org/els/health-systems/Table-of-Content-Metadata-OECD-Health-Statistics-2020.pdf

[4] OECD. Health at a glance 2021, OECD Indicators. Paris: OECD Publishing; 2021. 10.1787/ae3016b9-en

[5] Bolczek C, Nimptsch U, Möckel M, Mansky T. Health care provision and volume-outcome relationship for acute myocardial infarction: long-term analysis of german nationwide hospital discharge data 2005–2015. Gesundheitswesen. 2020;82(10):777–85. 10.1055/a-0829-6580 30822816

[6] OECD/European Observatory on Health Systems and Policies (2019). State of Health in the EU. Germany. Country Health Profile 2019.

[7] Nimptsch U, Mansky T. G-IQI | German Inpatient Indicators Version 5.3. Bundesreferenzwerte für das Auswertungsjahr 2019. Working Papers in Health Services Research Vol 4. Berlin: Universitätsverlag der Technischen Universität Berlin; 2021. 10.14279/depositonce-12342

[8] Kuch B, Bolte HD, Hoermann A, Meisinger C, Loewel H (2002). What is the real hospital mortality from acute myocardial infarction? Epidemiological vs clinical view. Eur Heart J.

[9] Vallabhajosyula S, Dunlay SM, Bell MR, Miller PE, Cheungpasitporn W, Sundaragiri PR, Kashani K, Gersh BJ, Jaffe AS, Holmes DR, Barsness GW (2020). Epidemiological trends in the timing of In-Hospital death in Acute Myocardial infarction – cardiogenic shock in the United States. J Clin Med.

[10] Sozialgericht Dresden. Urteil vom 24.02.2005, S 18 KR 180/02. München: Sozialgerichtsbarkeit Bundesrepublik Deutschland. Accessed 13 Oct 2023. https://www.sozialgerichtsbarkeit.de/legacy/23973?modul=esgb&id=23973

[11] Landessozialgericht Rheinland-Pfalz. Urteil vom 09.07.2020, L 5 KR 154/19. Mainz: Rechtsanwalt Friedrich W. Mohr. Accessed 13. Oct 2023. https://www.medizinrecht-ra-mohr.de/pdfs/Urteil_LSG-RLP_L_5_KR_154_19.pdf

[12] Nimptsch U, Busse R, Möckel M (2023). Recording early deaths following emergency department visits in inpatient data – observational study using data of 16 German hospitals. Z Evid Fortbild Qual Gesundhwes.

[13] Centers for Medicare & Medicaid Services (CMS). Fact Sheet: Two-Midnight Rule. Baltimore, MD: Centers for Medicare & Medicaid Services. Accessed 13. Oct 2023. https://www.cms.gov/newsroom/fact-sheets/fact-sheet-two-midnight-rule-0

[14] Hirsch R. Oct. Should treatment of acute MI be inpatient? Outpatient? The codes, the rules, and the money. St. Paul, MN: RAC Monitor. Accessed 13 2023. https://racmonitor.com/should-treatment-of-acute-mi-be-inpatient-outpatient-the-codes-the-rules-and-the-money/

[15] Cohen RA, Terlizzi EP, Cha AE et al. National Center for Health Statistics. Health insurance coverage: early release of estimates from the national health interview survey, 2020. Baltimore, MD: Centers for Medicare & Medicaid Services. 10.15620/cdc:108816

[16] Wilson M, Cutler D (2014). Emergency department profits are likely to continue as the Affordable Care Act expands coverage. Health Aff (Millwood).

[17] Research Data Centers of the Federal Statistical Office and Statistical Offices of the Federal States. DRG statistics. 10.21242/23141.2014.00.00.1.2.1.2019.00.00.1.2.1, own calculations.

[18] Agency for Healthcare Research and Quality. HCUP National Inpatient Sample (NIS). Healthcare Cost and Utilization Project (HCUP) http://www.hcup-us.ahrq.gov/nisoverview.jsp (2022). Accessed 27 Feb 2024.

[19] Agency for Healthcare Research and Quality. HCUP Nationwide Emergency Department Sample (NEDS). Healthcare Cost and Utilization Project (HCUP) https://www.hcup-us.ahrq.gov/nedsoverview.jsp (2022). Accessed 27 Feb 2024.

[20] Organisation for Economic Co-operation and Development (OECD). Health Care Quality and Outcomes (HCQO) 2020-21 Data Collection. Paris: OECD. Accessed 13 Oct 2023. https://www.oecd.org/els/health-systems/Definitions-of-Health-Care-Quality-Outcomes.pdf

[21] Krumholz HM, Wang Y, Mattera JA (2006). An administrative claims model suitable for profiling hospital performance based on 30-day mortality rates among patients with an acute myocardial infarction. Circulation.

[22] Organisation for Economic Co-operation and Development (OECD). Health Care Quality and Outcomes (HCQO) 2018-19 Data Collection. Paris: OECD. Accessed 13 Oct 2023. https://www.oecd.org/statistics/data-collection/Health Care Quality Indicators_guidelines.pdf

[23] Nimptsch U, Busse R (2021). [ST-elevation myocardial infarction and percutaneous coronary intervention: analysis of Time Stamps in Hospital Administrative Data]. Gesundheitswesen.

[24] Houchens R, Ross D, Elixhauser A. Final Report on Calculating National Inpatient Sample (NIS) Variances for Data Years 2012 and Later. HCUP Methods Series Report # 2015-09 ONLINE. Rockville, MD: U.S. Agency for Healthcare Research and Quality 2015. Accessed 27 Feb 2024. http://www.hcup-us.ahrq.gov/reports/methods/methods.jsp

[25] Benchimol EI, Smeeth L, Guttmann A, the RECORD Working Committee (2015). The REporting of studies conducted using Observational routinely-collected health data (RECORD) Statement. PLoS Med.

[26] Stolpe S, Kowall B, Werdan K, Zeymer U, Bestehorn K, Weber MA, Schneider S, Stang A (2023). OECD indicator ‘AMI 30-day mortality’ is neither comparable between countries nor suitable as indicator for quality of acute care. Clin Res Cardiol.

[27] Carinci F, Van Gool K, Mainz J, OECD Health Care Quality Indicators Expert Group (2015). Towards actionable international comparisons of health system performance: expert revision of the OECD framework and quality indicators. Int J Qual Health Care.

[28] André R, Bongard V, Elosua R (2014). International differences in acute coronary syndrome patients’ baseline characteristics, clinical management and outcomes in Western Europe: the EURHOBOP study. Heart.

[29] Bottle A, Middleton S, Kalkman CJ, Livingston EH, Aylin P (2013). Global comparators project: international comparison of hospital outcomes using administrative data. Health Serv Res.

[30] Maier B, Loewe A, Larscheid P (2021). [Out-of-hospital and in-hospital death from myocardial infarction in Berlin]. Gesundheitswesen.

[31] Goldberg RJ, Glatfelter K, Burbank-Schmidt E (2006). Trends in community mortality due to coronary heart disease. Am Heart J.

[32] OECD. National area distribution (indicator). 2024. 10.1787/34f4ec4a-en

[33] Ebeling M, Mühlichen M, Talbäck M (2024). Disease incidence and not case fatality drives the rural disadvantage in myocardial-infarction-related mortality in Germany. Prev Med.

[34] Groene O, Kristensen S, Arah OA, DUQuE Project Consortium (2014). Feasibility of using administrative data to compare hospital performance in the EU. Int J Qual Health Care.

[35] Heber R, Levsen A, Offermanns M. Meaningfulness of hospital structure and quality comparisons based on OECD data. [Aussagekraft von Krankenhausstruktur- und Qualitätsvergleichen auf Basis von OECD-Daten. Düsseldorf: Deutsches Krankenhausinstitut; 2021. Accessed Feb 27 2024. https://www.dkgev.de/fileadmin/default/Mediapool/1_DKG/1.7_Presse/1.7.1_Pressemitteilungen/2021/20210701_Endbericht_OECD-Daten_DKI.PDF

