# Supplementary material for: Impact of early death recording on international comparison of acute myocardial infarction mortality – administrative hospital data study using the example of Germany and the United States
Source: BMC Health Serv Res. 2024 May 7;24:593. doi: 10.1186/s12913-024-11044-6 (PMC11075306; doi:10.1186/s12913-024-11044-6)

Impact of early death recording on international comparison of acute myocardial infarction mortality – Administrative hospital data study using the example of Germany and the United States

Supplementary material

Supplementary material 1 Definition of study variables

	**Germany**		**United States**	
	DRG statistics		National Inpatient Sample (NIS) and Nationwide Emergency Department Sample (NEDS)	
	**Inclusion**	**Exclusion**	**Inclusion**	**Exclusion**
**Unit of analysis**				
Treatment for acute myocardial infarction	PD ICD-10-GM I21 I22	Age < 15	PD ICD-10-CM I21 I22; PD ICD-9-CM 410	Age < 15
**Clinical presentation and severity**				
Transmural/ST-elevation myocardial infarction (STEMI)	PD ICD-10-GM I210 I211 I212 I213		PD ICD-10-CM I210 I211 I212 I213; PD ICD-9-CM 4100 4101 4102 4103 4104 4105 4106 4108	
Cardiogenic shock	SD ICD-10-GM R570		SD ICD-10-CM R570; SD ICD-9-CM 78551	
Resuscitation	OPS 8771		ICD-9-CM 9960; ICD-10-PCS 5A12012; CCS 92950	
**Revascularization**				
Percutaneous coronary intervention (without coronary surgery)	OPS 88370 88371 8837288375 88376 88377 88378 88379 8837k 8837m 8837p 8837q 8837t 8837u 8837v 8837w 88399 883d0 883d1 883d2	OPS 5361 5362 5363	ICD-9-CM 0066 3601 3602 3605 ICD-10-PCS 02703 02704 02713 02714 02723 02724 02733 02734; CCS 45	ICD-9-CM 3610 3611 3612 3613 3614 3615 3616 3617 3619; ICD-10-PCS 02100 02110 02120 02130; CCS 44
Coronary artery bypass graft	OPS 5361 5362 5363		ICD-9-CM 3610 3611 3612 3613 3614 3615 3616 3617 3619; ICD-10-PCS 02100 02110 02120 02130; CCS 44	

PD: principal or first listed diagnosis, SD: secondary diagnosis, ICD: international classification of diseases, GM: German modification, CM: clinical modification, OPS: German procedure coding system, PCS: procedure coding system, CCS: Clinical Classifications Software services and procedures classification.

Supplementary material 2 Selection of study population, accumulated data of 2014 to 2019

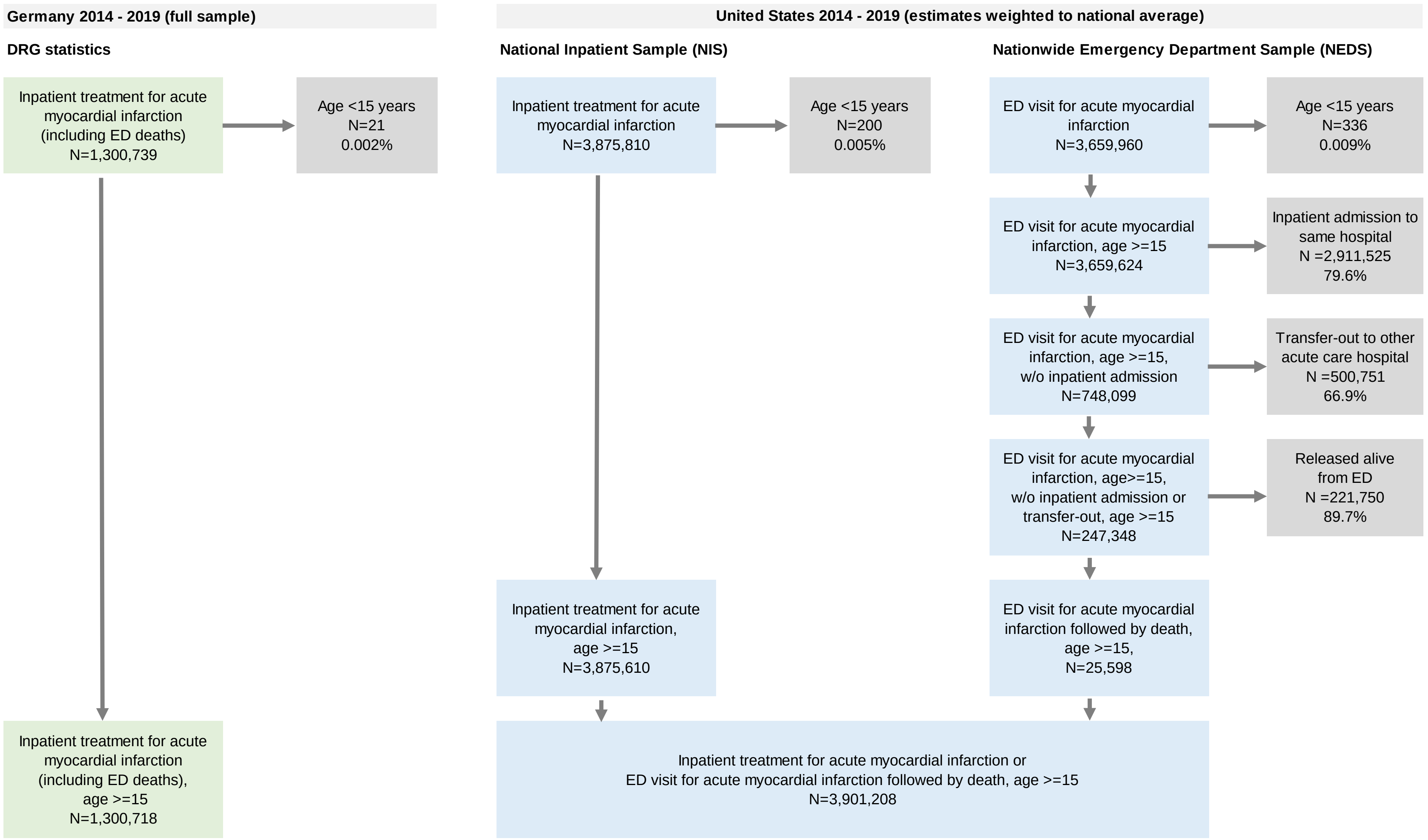

Supplementary material 3 Characteristics of cases treated for acute myocardial infarction by year, 2014-2019

	Year	Germany (full sample)	United States (estimates weighted to national average)		
		DRG statistics	National Inpatient Sample (NIS)	Nationwide Emergency Department Sample (NEDS)	NIS and NEDS combined
		Inpatient treatment (including ED deaths)	*Inpatient treatment*	*ED death without admission*	*Inpatient treatment or ED death without admission*
Cases with a principal/first listed diagnosis of AMI (age>=15) N	2014	219,546	608,760	4,790	613,550
	2015	219,248	629,890	4,981	634,871
	2016	219,229	651,189	4,162	655,351
	2017	217,715	662,075	3,924	665,998
	2018	212,350	658,545	3,763	662,309
	2019	212,630	665,150	3,978	669,128
Cases per 100,000 population (age-and-sex standardized) [a]	2014	270.4 (245.6)	190.9 (232.1)	1.5 (1.8)	192.4 (233.9)
	2015	266.8 (241.7)	196.0 (235.3)	1.5 (1.9)	197.5 (237.1)
	2016	265.7 (238.9)	201.5 (239.0)	1.3 (1.5)	202.8 (240.5)
	2017	263.0 (234.6)	203.3 (237.9)	1.2 (1.4)	204.5 (239.3)
	2018	255.8 (226.8)	201.3 (232.5)	1.2 (1.3)	202.4 (233.8)
	2019	255.7 (224.5)	202.6 (231.0)	1.2 (1.4)	203.9 (232.4)
Female N (%)	2014	74,970 (34.1)	232,325 (38.2)	1,996 (41.7)	234,322 (38.2)
	2015	74,132 (33.8)	240,905 (38.2)	2,017 (40.5)	242,922 (38.3)
	2016	73,333 (33.5)	247,450 (38.0)	1,686 (40.5)	249,136 (38.0)
	2017	72,217 (33.2)	251,830 (38.0)	1,673 (42.6)	253,503 (38.1)
	2018	69,620 (32.8)	247,125 (37.5)	1,523 (40.5)	248,648 (37.5)
	2019	69,258 (32.6)	247,950 (37.3)	1,658 (41.7)	249,608 (37.3)
Age >= 65 years N (%)	2014	143,810 (65.5)	344,140 (56.5)	3,198 (66.8)	347,338 (56.6)
	2015	143,751 (65.6)	357,625 (56.8)	3,526 (70.8)	361,151 (56.9)
	2016	142,557 (65.0)	371,115 (57.0)	2,900 (69.7)	374,015 (57.1)
	2017	141,391 (64.9)	377,885 (57.1)	2,720 (69.3)	380,605 (57.1)
	2018	137,583 (64.8)	376,615 (57.2)	2,626 (69.8)	379,241 (57.3)
	2019	138,022 (64.9)	382490 (57.5)	2889 (72.6)	385379 (57.6)
Age Median (P25 - P75)	2014	72 (60 - 80)	66 (56 - 77)	72 (60 - 83)	66 (56 - 77)
	2015	72 (60 - 80)	67 (57 - 77)	73 (62 - 84)	67 (57 - 77)
	2016	72 (60 - 80)	67 (57 - 77)	72 (62 - 83)	67 (57 - 77)
	2017	71 (60 - 80)	67 (57 - 77)	71 (61 - 82)	67 (57 - 77)
	2018	71 (60 - 80)	67 (57 - 77)	72 (62 - 82)	67 (57 - 77)
	2019	71 (60 - 80)	67 (57 - 76)	74 (63 - 84)	67 (57 - 76)

Supplementary material 3 (continued)

	Year	Germany (full sample)	United States (estimates weighted to national average)		
		DRG statistics	National Inpatient Sample (NIS)	Nationwide Emergency Department Sample (NEDS)	NIS and NEDS combined
		Inpatient treatment (including ED deaths)	*Inpatient treatment*	*ED death without admission*	*Inpatient treatment or ED death without admission*
Transmural/ST-elevation AMI N (%)	2014	74,702 (34.0)	151,285 (24.9)	1,226 (25.6)	152,511 (24.9)
	2015	71,870 (32.8)	158,755 (25.2)	2,061 (41.4)	160,816 (25.3)
	2016	70,484 (32.2)	176,415 (27.1)	3,560 (85.5)	179,975 (27.5)
	2017	69,383 (31.9)	173,485 (26.2)	2,962 (75.5)	176,447 (26.5)
	2018	68,734 (32.4)	168,550 (25.6)	2,024 (53.8)	170,574 (25.8)
	2019	67,460 (31.7)	172,995 (26.0)	2,268 (57.0)	175,263 (26.2)
Cardiogenic shock N (%)	2014	13,667 (6.2)	36,435 (6.0)	448 (9.3)	36,883 (6.0)
	2015	13,972 (6.4)	37,725 (6.0)	470 (9.4)	38,195 (6.0)
	2016	14,175 (6.5)	39,480 (6.1)	405 (9.7)	39,885 (6.1)
	2017	14,689 (6.7)	40,610 (6.1)	394 (10.0)	41,004 (6.2)
	2018	14,618 (6.9)	42,280 (6.4)	380 (10.1)	42,660 (6.4)
	2019	14,137 (6.6)	43,600 (6.6)	331 (8.3)	43,931 (6.6)
Resuscitation N (%)	2014	12,118 (5.5)	11,470 (1.9)	2,476 (51.7)	13,946 (2.3)
	2015	12,185 (5.6)	11,735 (1.9)	2,487 (49.9)	14,222 (2.2)
	2016	12,178 (5.6)	11,530 (1.8)	2,141 (51.5)	13,671 (2.1)
	2017	12,112 (5.6)	11,855 (1.8)	2,120 (54.0)	13,975 (2.1)
	2018	11,741 (5.5)	11,925 (1.8)	1,982 (52.7)	13,907 (2.1)
	2019	11,464 (5.4)	13,125 (2.0)	2,106 (52.9)	15,231 (2.3)
Percutaneous coronary intervention N (%)	2014	122,222 (55.7)	287,470 (47.2)	287 (6.0)	287,757 (46.9)
	2015	124,489 (56.8)	296,425 (47.1)	273 (5.5)	296,698 (46.7)
	2016	128,586 (58.7)	306,095 (47.0)	285 (6.9)	306,380 (46.8)
	2017	129,821 (59.6)	311,030 (47.0)	310 (7.9)	311,340 (46.7)
	2018	129,419 (60.9)	313,440 (47.6)	284 (7.5)	313,724 (47.4)
	2019	131,960 (62.1)	320,980 (48.3)	274 (6.9)	321,254 (48.0)
Coronary artery bypass graft N (%)	2014	12,166 (5.5)	52,820 (8.7)	<=10 (<=0.1)	<=52,830 (<=8.6)
	2015	12,042 (5.5)	54,815 (8.7)	0 (0.0)	54,815 (8.6)
	2016	12,174 (5.6)	56,945 (8.7)	0 (0.0)	56,945 (8.7)
	2017	12,139 (5.6)	57,420 (8.7)	0 (0.0)	57,420 (8.6)
	2018	11,608 (5.5)	57,420 (8.7)	0 (0.0)	57,420 (8.7)
	2019	11,468 (5.4)	59,450 (8.9)	0 (0.0)	59,450 (8.9)

Supplementary material 3 (continued)

	Year	Germany (full sample)	United States (estimates weighted to national average)		
		DRG statistics	National Inpatient Sample (NIS)	Nationwide Emergency Department Sample (NEDS)	NIS and NEDS combined
		Inpatient treatment (including ED deaths)	*Inpatient treatment*	*ED death without admission*	*Inpatient treatment or ED death without admission*
Transferred-in from other acute care hospital N (%)	2014	31,902 (14.5)	107,265 (17.5)	n/a	107,265 (17.5)
	2015	31,319 (14.3)	109,810 (17.3)	n/a	109,810 (17.3)
	2016	30,144 (13.8)	113,625 (17.4)	n/a	113,625 (17.3)
	2017	29,397 (13.5)	118,665 (17.9)	n/a	118,665 (17.8)
	2018	27,241 (12.8)	120,705 (18.3)	n/a	120,705 (18.2)
	2019	25,695 (12.1)	125,150 (18.8)	n/a	125,150 (18.7)
Treated in emergency department N (%)	2014	n/a	425,110 (69.8)	4,790 (100.0)	429,900 (70.1)
	2015	n/a	445,075 (70.7)	4,981 (100.0)	450,056 (70.9)
	2016	n/a	464,840 (71.4)	4,162 (100.0)	469,002 (71.6)
	2017	n/a	480,645 (72.6)	3,924 (100.0)	484,568 (72.8)
	2018	n/a	494,000 (75.0)	3,763 (100.0)	497,764 (75.2)
	2019	n/a	502,265 (75.5)	3,978 (100.0)	506,243 (75.7)
Same day stay N (%) [b]	2014	14,097 (6.4)	18,010 (3.0)	4,790 (100.0)	22,800 (3.7)
	2015	14,163 (6.5)	18,370 (2.9)	4,981 (100.0)	23,351 (3.7)
	2016	14,125 (6.4)	19,700 (3.0)	4,162 (100.0)	23,862 (3.6)
	2017	13,919 (6.4)	20,655 (3.1)	3,924 (100.0)	24,579 (3.7)
	2018	13,124 (6.2)	19,860 (3.0)	3,763 (100.0)	23,623 (3.6)
	2019	12,605 (5.9)	21,415 (3.2)	3,978 (100.0)	25,393 (3.8)
Length of stay Median (P25 - P75)	2014	6 (3 - 10)	2 (1 - 5)	n/a	2 (1 - 5)
	2015	6 (3 - 9)	2 (1 - 5)	n/a	2 (1 - 5)
	2016	6 (3 - 9)	2 (1 - 5)	n/a	2 (1 - 5)
	2017	6 (3 - 9)	2 (1 - 5)	n/a	2 (1 - 5)
	2018	5 (3 - 9)	2 (1 - 5)	n/a	2 (1 - 5)
	2019	5 (3 - 9)	2 (1 - 5)	n/a	2 (1 - 5)
Transferred-out to other acute care hospital N (%)	2014	39,145 (17.8)	49,550 (8.1)	n/a	49,550 (8.1)
	2015	38,229 (17.4)	50,170 (7.9)	n/a	50,170 (7.9)
	2016	37,036 (16.9)	50,875 (7.8)	n/a	50,875 (7.8)
	2017	36,125 (16.6)	50,905 (7.7)	n/a	50,905 (7.6)
	2018	33,517 (15.8)	48,905 (7.4)	n/a	48,905 (7.4)
	2019	31,676 (14.9)	48,275 (7.3)	n/a	48,275 (7.2)

Supplementary material 3 (continued)

	Year	Germany (full sample)	United States (estimates weighted to national average)		
		DRG statistics	National Inpatient Sample (NIS)	Nationwide Emergency Department Sample (NEDS)	NIS and NEDS combined
		Inpatient treatment (including ED deaths)	*Inpatient treatment*	*ED death without admission*	*Inpatient treatment or ED death without admission*
In-hospital death N (%)	2014	18,853 (8.6)	30,315 (5.0)	4,790 (100.0)	35,105 (5.7)
	2015	18,680 (8.5)	30,205 (4.8)	4,981 (100.0)	35,186 (5.5)
	2016	17,858 (8.1)	30,700 (4.7)	4,162 (100.0)	34,862 (5.3)
	2017	17,936 (8.2)	31,035 (4.7)	3,924 (100.0)	34,959 (5.2)
	2018	17,533 (8.3)	30,150 (4.6)	3,763 (100.0)	33,913 (5.1)
	2019	16,881 (7.9)	29,935 (4.5)	3,978 (100.0)	33,913 (5.1)
In-hospital death within 30 days N (%)	2014	18,307 (8.3)	29,905 (4.9)	4,790 (100.0)	34695 (5.7)
	2015	18,146 (8.3)	29,760 (4.7)	4,981 (100.0)	34741 (5.5)
	2016	17,384 (7.9)	30,265 (4.6)	4,162 (100.0)	34,427 (5.3)
	2017	17,406 (8.0)	30,620 (4.6)	3,924 (100.0)	34,544 (5.2)
	2018	17,106 (8.1)	29,725 (4.5)	3,763 (100.0)	33,488 (5.1)
	2019	16,482 (7.8)	29,455 (4.4)	3,978 (100.0)	33,433 (5.0)

AMI: acute myocardial infarction; ED: emergency department; n/a: not available. [a] Directly standardized by sex and 5-year age-groups according to the 2010 OECD standard population, age >=15. [b] As in US NEDS data no information on length of stay is available for ED visits without inpatient admission, ED deaths without admission were assigned to a length of stay <1 day (same-day stay).

Supplementary material 4 30-day AMI in-hospital mortality stratified by patient and treatment characteristics, accumulated data of 2014 to 2019

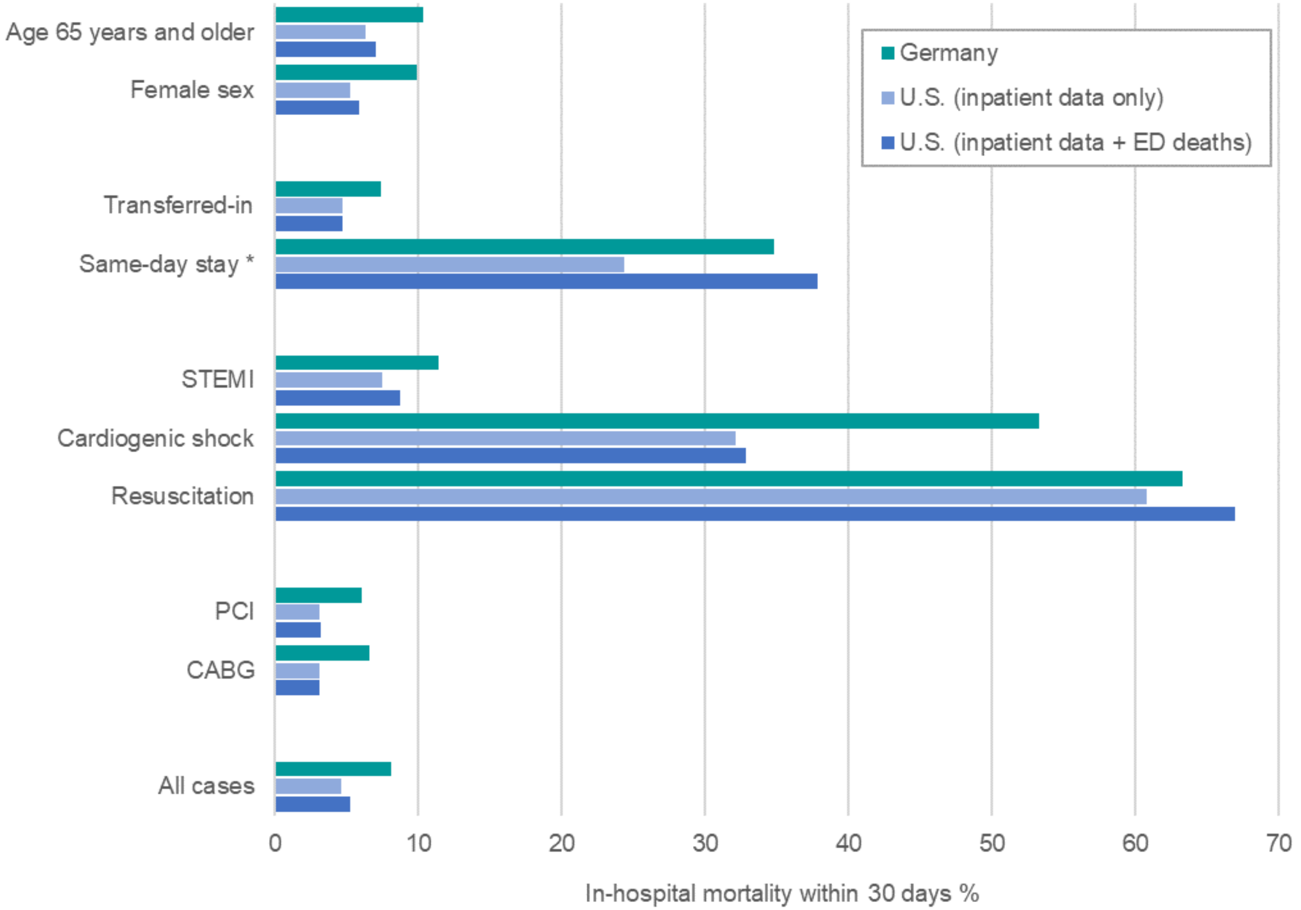

Supplement: Supplementary file 1 — Supplementary Material 1 [file 12913_2024_11044_MOESM1_ESM.pdf]
